# A Review on the Research Progress of Imprint Film Materials for Nanoimprint Lithography

**DOI:** 10.3390/mi17050596

**Published:** 2026-05-13

**Authors:** Zhiwei Yang, Rui Ma, Chuangye Yao, Jinsong Song, Jingrun Li, Guangxu Cui, Haiming Li, Yuanxun Cao, Dayong Ma

**Affiliations:** 1 School of Intelligent Manufacturing, Jilin Vocational College of Industry and Technology, Jilin 132013, China; 2 School of Materials Science and Engineering, North University of China, Taiyuan 030051, China; 3 School of Physics and Electronic Electrical Engineering, Xiangnan University, Chenzhou 423000, China; 4 School of Mechanical Engineering, Hebei Petroleum University of Technology, Chengde 067000, China; 5 School of Intelligent Manufacturing, Jilin General Aviation Vocational and Technical College, Jilin 132211, China

**Keywords:** nanoimprint lithography, imprint film material, single polymer, composite structure, transparent electrode

## Abstract

Nanoimprint lithography (NIL) is highly dependent on imprinted film as a pattern-transfer medium. This paper systematically reviews the research progress of imprint film materials for NIL. Firstly, polydimethylsiloxane (PDMS), polyethylene terephthalate (PET), polyvinyl alcohol (PVA) and other single-polymer films are discussed, and their respective advantages (such as low surface energy, high optical transparency, water solubility) and inherent limitations (elastic deformation, demolding difficulties, humidity sensitivity)) are summarized. In order to overcome the above contradiction, researchers developed a composite imprint film structure, including an elastomer–rigid bilayer template and sandwich structure film, which achieved high resolution, conformal contact and facile demolding characteristics through mechanical function decoupling. At the same time, the emerging polymer/transparent electrode composite system (such as AgNWs/PVA, AgNWs/PDMS) gives the film active functions such as self-heating and antistatic ones, which effectively solves the key challenges in thermal management and electrostatic control. This paper comprehensively presents the evolution path from single-material to multi-functional composites, and provides guidance for the design of advanced imprint film for high precision, high reliability and large-scale NIL applications.

## 1. Introduction

Nanoimprint lithography has become one of the key technologies in the field of micro nano processing due to its advantages of high resolution, high throughput and low cost [1,2,3,4,5]. In this technology system, the imprint film is the medium for the definition and transfer of graphics, and its material properties directly determine the fidelity of the pattern, the reliability of the process and the performance of the final device [6,7,8,9,10,11,12,13,14,15]. With the expansion of nanoimprint lithography applications from traditional planar substrates to flexible electronics, three-dimensional functional structures and optoelectronic devices [16,17,18,19,20], the requirements for imprint film materials have evolved from a single structural support function to multiple requirements for mechanical properties, optical transparency, surface properties and functionality [20,21,22,23,24,25,26,27,28,29,30]. This evolution has prompted researchers to conduct in-depth research on the limitations of traditional imprint film materials and continue to explore new materials.

The development of thin film materials used in nanoimprint lithography shows that single-polymer is still the key to determine the replication accuracy, durability and continuous manufacturing ability [30,31,32,33,34,35,36,37,38,39,40,41]. PDMS has low surface energy, high UV transmission and controllable elastic modulus to achieve excellent conformal contact [42,43,44,45,46,47,48]; it can be applied to patterned sapphire substrates and flexible devices [49,50,51,52]. However, in its repeated use, the surface Young’s modulus increases and deteriorates due to the diffusion of low molecular weight resist to the network and in situ crosslinking [53,54,55,56]. PET has become the mainstream substrate for thermal nanoimprinting and roll-to-roll (R2R) UV-NIL due to its excellent viscoelastic flow above Tg, high light transmittance, flexibility and low cost [57,58,59]. On the one hand, PVA is used as a water-soluble sacrificial mold to realize stress-free demolding and prepare dielectric superlens with an aspect ratio of up to 5.8 [60,61]. However, due to the lack of single-layer etching resistance, achieving metal stripping for small linewidth requires building a double-layer inverted cone profile with hydrogen silsesquioxane (HSQ) [62]. On the other hand, as a humidity responsive hydrogel, a Fabry–Perot cavity with a volume swelling rate of ≈ 62.5% is constructed, which realizes the millisecond RGB (red, green, blue) full-color pixels, and has the potential for both dynamic display and humidity sensing [63,64,65].

Although the above-mentioned single-polymer materials show unique advantages in their respective application fields, their inherent limitations are also increasingly prominent, creating an inherent trade-off that is difficult to resolve within a single material [34]. The elastic materials represented by PDMS, with low Young’s modulus and excellent flexibility, have achieved good conformal contact and facile demolding characteristics [66,67,68]. However, their elastic nature leads to lateral expansion under pressure, which leads to pattern distortion, and limits their application in sub-100 nm high-precision machining [69,70]. Although the rigid materials represented by PET and PVA can achieve high-precision graphics transfer in an ideal ratio of 1:1, their strong adhesion to the substrate after curing leads to difficult peeling and lack of buffer capacity [71,72,73,74,75,76,77], which very easily causes damage to brittle or precision substrates. This performance contradiction between “elasticity and deformation” and “hardness and peeling” constitutes a technical bottleneck for a single polymer material in the application of higher precision and more complex structures.

In order to break through the performance limitations of a single material, researchers have turned to the composite and structural design strategy, aiming to realize the integration and optimization of multiple properties through the synergy and functional complementarity of different materials [78,79,80,81,82]. The elastic rigid double-layer composite template separates the graphical function from the mechanical adaptation function, provides the conformal contact ability with the elastic support layer, ensures the accurate imprinting of sub-15 nanometer high-resolution graphics with the ultra-thin high cross-linked rigid structure layer, and solves the key problem of easy delamination of the two-layer interface due to the large difference in modulus through the polymer interpenetrating network structure bonding technology [83,84,85]. Furthermore, the sandwich structure composite imprint film is introduced into the middle layer to decouple the lateral expansion of the elastic layer and the deformation of the imprint layer [86,87,88,89,90]. The deformation problem of a single elastic material and the stripping problem of a single flexible material are successfully solved through the absorption of external forces by the upper protective layer, the resistance of the middle rigid substrate to lateral tension, and the transfer of high fidelity graphics and easy stripping by the lower elastic layer [34].

In recent years, the research of imprint film materials is moving from passive structural adaptation to active functional integration [91,92,93]. It has become a cutting-edge direction in this field to composite functional transparent electrode materials (such as silver nanowires) with a polymer matrix to construct multilayer or embedded structures [94,95,96,97,98,99,100]. In terms of thermal management, the self-heating composite film based on AgNWs/PVA uses the Joule heating effect to realize rapid and uniform heating of the imprinted film itself (heating to 60 °C within 30 s under 10 V voltage) [101,102]. Its heating and cooling rate is much higher than that of the traditional hot plate [103,104,105,106,107,108,109,110]. At the same time, the shrinkage effect of the PVA matrix should promote the “nano cold welding” between silver nanowires (AgNWs), and reduce the sheet resistance to 10 Ω/sq while maintaining the 248 nm UV transmittance of 84.82% [101]. In terms of electrostatic control, the AgNWs/PDMS three-dimensional transparent conductive film embeds the conductive network into the PDMS surface, which improves the effective conductivity of the composite by several orders of magnitude, and reduces the charge relaxation time [111,112,113,114,115,116,117,118,119,120,121,122,123,124,125]. From the range of minutes to hours of pure PDMS to the millisecond level, real-time electrostatic neutralization is realized in the imprinting process, and electrostatic defects are significantly reduced [126,127,128,129]. This kind of polymer/transparent electrode composite system endows the imprint film with self-heating, antistatic and other key properties through the coordination of the functional phase and the matrix phase [130,131,132,133,134,135,136,137,138,139,140,141,142], opening up a new path for the development of the next generation of high-performance, multifunctional nano imprint film materials [143,144,145,146,147,148,149,150,151,152].

In summary, the research of thin film materials used in nanoimprint lithography is undergoing a profound evolution from single-material to composite structure, from passive adaptation to functional integration. This paper aims to systematically review the research progress in this field, and describe the application status, performance advantages and inherent limitations of PDMS-based, PET-based and PVA-based imprinted films. The design idea, mechanical mechanism and performance characteristics of the elastic rigid double-layer composite template and the sandwich structure composite imprinting film were analyzed. The function integration effect of the polymer/transparent electrode composite system in frontier fields such as thermal management and electrostatic control is discussed. Through the integration and analysis of the above research context, this paper aims to reveal the internal logic and key challenges of the development of imprint film materials, and provide reference for the design of advanced materials for high-precision, high-reliability and multifunctional nanoimprint applications.

## 2. Single-Material Nanoimprint Film

### 2.1. Research Progress on the Application of PDMS-Based Nanoimprint Film

In the nano-imprint lithography technology system, polydimethylsiloxane has become one of the most widely used soft stamp materials due to its unique physical and chemical properties [153,154]. PDMS has a low surface energy (about 20–22 Mn/M), which can achieve good demolding separation with a variety of UV-curable photoresists [155]. Generally, no additional anti-adhesion layer is required, which simplifies the process and reduces the risk of pollution. PDMS has excellent transmittance in a UV band, which makes it especially suitable for UV nanoimprinting processes [156,157,158]. The inherent flexibility and controllable elastic modulus of PDMS enable it to achieve conformal contact on uneven or even curved substrates, which is particularly important for the fabrication of graphical sapphire substrates, flexible electronic devices and large-area solar cells [159]. However, with the accumulation of imprinting times, the performance degradation of the PDMS seal has become increasingly prominent [160], which has become one of the key factors restricting its industrial application.

Aiming at the degradation mechanism of the PDMS nanoimprint film, researchers systematically studied the evolution law of the material hardness of the nanoimprint film with the number of imprints by using the force distance measurement technology of an atomic force microscope [161]. It was found that the Young’s modulus of the seal surface shows a significant upward trend with the increase in imprinting times. The fundamental reason for this phenomenon is that low molecular weight resist components (such as monomers, oligomers or photoinitiators) diffuse into the PDMS molecular network during the imprinting process, and crosslink in situ under UV irradiation or thermal action, thus changing the mechanical properties of the PDMS bulk [162]. By comparing two acrylate photoresists with different average molecular weights (LR 8,996,420 g/mol; LR po84f, 1590 g/mol), the study showed that the hardening rate of the stamp surface of low-molecular-weight photoresist was significantly higher than that of high-molecular-weight photoresist due to its faster diffusion rate [161]. Based on this understanding of the mechanism, the study further used epoxy resin epoxy-based negative photoresist (SU-8) with higher molecular weight (about 7000 g/mol) and complex aromatic ring structure as a resist, combined with the temperature-assisted UV imprinting process, which successfully increased the service life of the nano imprint film to more than 100 imprints without a measurable hardness change being detected (as shown in Table 1) [161]. This work not only reveals the internal relationship between the resist molecular structure and seal life, but also provides a clear design direction for the performance optimization of the PDMS seal.

**Figure 1:**
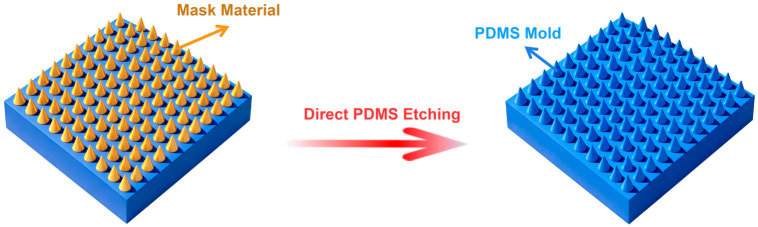
Structural diagram of large-area self-assembly mask and PDMS direct etching technology [163].

In terms of the preparation method of the PDMS seal, researchers have proposed a new method that breaks through the traditional molding [163]. Traditional PDMS mold manufacturing depends on the replication of silicon master, and the minimum feature size is limited by the filling ability of PDMS in the master micro nano structure, which usually requires the use of low-viscosity PDMS or a diluted solvent, but this kind of material often has poor mechanical properties, which limits the number of seals to be reused [164,165]. To solve this contradiction, the self-assembled polystyrene nanoparticle monolayer was introduced as an etching mask in this study, and the cured PDMS was directly patterned by oxygen plasma dry etching, as shown in Figure 1 [163]. Because the etching process is independent of the viscosity of PDMS, this method can achieve high-resolution structure preparation with a characteristic size of about 100 nm without sacrificing the mechanical properties of the material [166]. The PDMS seal prepared by this method successfully replicates the large-area plasmon super surface structure, and verifies its feasibility in the manufacture of sub wavelength optical devices [167]. This technology path provides a new idea for the preparation of a high-precision and high-durability soft seal.

**Figure 2:**
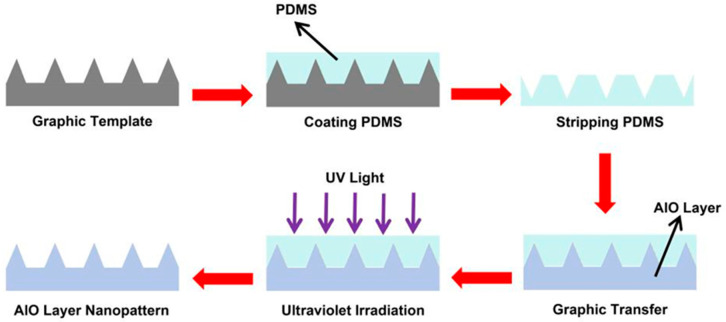
Preparation of PDMS nanoimprint film and transfer of the nanopattern to the surface of alumina doped with UV curing agent layer [168].

In terms of application expansion, PDMS-based nanoimprint films have extended from the traditional field of micro nano structure replication to the manufacture of functional optoelectronic devices. It was found that the UV-curable polymer was mixed with the alumina precursor solution, and the one-dimensional linear nano grating structure was prepared on the glass substrate by using the PDMS template through the UV nanoimprint technology, as shown in Figure 2 [168]. As a liquid crystal orientation layer, the nano patterned film achieves uniform unidirectional orientation by inducing geometric deformation of liquid crystal molecules. Its pretilt angle can reach about 0.53°, and its transmittance is about 1.5% higher than that of the traditional polyimide orientation layer [168,169,170]. This work shows the potential application value of PDMS nanoimprint technology in the manufacturing of a liquid crystal display orientation layer, especially in flexible and crimpable display devices. Its non-contact and large-area process advantages are particularly prominent.

To sum up, the current research on PDMS-based nanoimprint films is developing from a single process replication technology to a systematic engineering direction covering degradation mechanism analysis, material process collaborative optimization and multifunctional device manufacturing. At the basic research level, it is expected to provide theoretical guidance for the design of high-life seals through in-depth understanding of the constitutive relationship between the resist molecular structure, diffusion behavior and the evolution of mechanical properties of seals. At the level of technology development, new mold preparation methods such as direct etching have opened up a new path to achieve the unity of high resolution and high durability. In terms of application expansion, PDMS nanoimprinting technology has gradually penetrated into cutting-edge fields such as graphical sapphire substrates, light-emitting diodes, super surface optical devices, biochips and flexible electronic devices, showing broad development prospects.

### 2.2. Research Progress on PET-Based Nanoimprint Film

Polyethylene terephthalate (PET), as an amorphous thermoplastic polymer, has become one of the most widely used thin film substrate materials in nano imprint lithography (NIL) technology due to its excellent viscoelastic flow behavior above the glass transition temperature (TG ≈ 80 °C), high optical transmittance (visible light transmittance of more than 87%), good mechanical flexibility, chemical stability and low cost [171,172]. In recent years, researchers have carried out systematic and in-depth work on the molding mechanism, process optimization and functional application of PET film in thermal nanoimprint (T-NIL) and UV-NIL [173].

In the field of thermal nanoimprinting (T-NIL), there is a strong nonlinear coupling relationship between the replication accuracy of PET film and the geometric characteristics and process parameters of mold microstructure [174,175,176]. The researchers creatively designed a star pattern with a continuous line width from 40 μm to 320 μm (as shown in Table 2), and systematically revealed the filling behavior and stress distribution of PET under different aspect ratios (0.3125–2.5) [171]. The finite element simulation results show that when the linewidth exceeds 240 μm (corresponding height width ratio < 0.417), the viscoelastic deformation ability of PET is enough to achieve complete filling, and the filling rate is up to 99% at 320 μm linewidth; when the aspect ratio exceeds 0.625 (linewidth < 160 μm), the filling effect deteriorates sharply, and the filling rate under 40 μm linewidth is only 19%. The study further pointed out that the molding quality of PET was highly sensitive to temperature. At 140 °C, the mobility of polymer segments is insufficient, resulting in missing imprint patterns and blurred edges. When the temperature rises to 160 °C, the material enters the optimized molding state of low modulus and fast relaxation, which can realize full-scale high fidelity replication from small linewidth (160 μm) to large linewidth (320 μm). However, when the temperature rises to 180 °C, excessive softening leads to the enhancement of surface adhesion, leading to a serious defect, which is that the pattern is torn off/lifted during demolding. In addition, the extension of holding time (from 400 s to 800 s) contributes significantly to the improvement of filling height and sidewall angle accuracy of a small linewidth structure, while the change in pressure in the range of 0.15–0.45 MPa has little effect on the replication accuracy of PET, showing its wide process window and good process robustness [171]. The study also revealed an important phenomenon: in the cooling and demolding stage, due to the release of residual stress, large linewidth structures (such as 320 μm) will have an obvious volume rebound, and the filling rate decreases by 10–17%, while the rebound effect of small linewidth structures (40 μm) is only 3%, reflecting the significant influence of size effect on the stability of demolded morphology [171].

**Figure 3:**
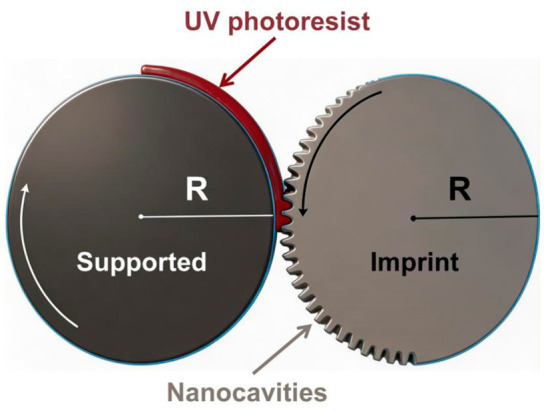
Schematic diagram of large-scale ultraviolet (UV) manufacturing of nano cavities [172].

In the field of roll-to-roll ultraviolet nanoimprinting (RTR-UV-NIL), PET, as a flexible substrate, shows unique advantages in large-area and continuous manufacturing. The researchers constructed a multiphase volume of fluid (VOF) numerical model combining the sliding mesh method and the open channel (OC) boundary conditions, and accurately simulated the transient behavior of UV resin filled with a nano column/pore structure on a PET substrate during the RTR-UV-NIL process for the first time, as shown in Figure 3 [172]. The model reveals that imprinting speed (IS) and resin viscosity are the two decisive parameters affecting the bubble entrapment defect. When the imprinting speed increases from 18.75 mm/s to 50 mm/s, the filling time shortens sharply, resulting in continuous bubble entrapment and nanostructure edge damage. When the viscosity of UV resin decreased from 200 CP to 180 CP, the wetting and filling effect of the resin on the nano cavity was significantly improved. Based on the simulation results, the researchers successfully prepared highly ordered and defect-free 300 nm elliptical nanorods and nanopore arrays on a PET film by corona treatment to enhance the surface polarity of PET, combined with the optimized process parameters (imprinting speed 18 mm/s, initial coating thickness 5 μm), and verified that the process still maintained good repeatability after continuous operation of 50 rolls (about 785.4 m), which provided a key process window for the industrial production of PET-based nanoimprint film [172,177,178].

**Figure 4:**
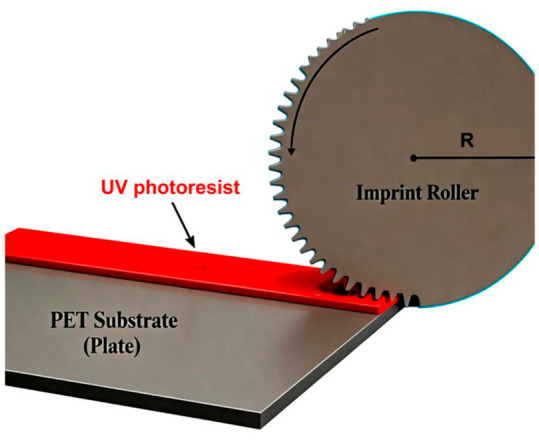
Schematic of the fabrication process on the flat substrate opposite the roller [179].

In terms of functional device integration, PET film has become an ideal choice for a flexible optoelectronic device substrate due to its excellent light transmittance, flexibility and low cost. Researchers combined RTR nanoimprint technology with a PET substrate to prepare non-fullerene organic solar cells (OSC). They used an R2P (roll-to-plate) nanoimprint to integrate light trapping nanostructures on a PET substrate, and verified the enhancement effect of the structure on light absorption through optical simulation, as shown in Figure 4 [179]. Compared with unstructured reference devices, devices with nanostructures show significant performance improvement: short-circuit current density (JSC) is increased by 15%, the fill factor (FF) is increased by 7%, and finally the power conversion efficiency (PCE) is improved by 25%, and the best device PCE is 6.5%, which is also the highest efficiency of indium tin oxide (ITO) free flexible PBDB-T:ITIC devices prepared by slit coating in an air environment using a non-toxic solvent reported at present [180,181]. This study not only verified the huge application potential of PET-based nanoimprint films in low-cost, green solvent-treated flexible photovoltaic devices, but also demonstrated the feasibility of roll-to-roll nanoimprint technology in high-throughput, large-area functional film manufacturing [179].

**Figure 5:**
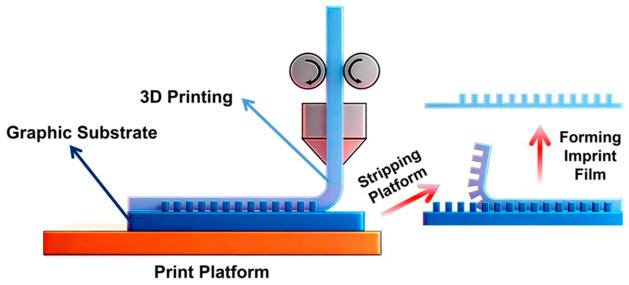
Schematic diagram of 3D-printing-assisted nanoimprint lithography [182].

At the same time, PET also shows the application potential as a high-precision functional template material. Researchers used a nanosecond-pulsed laser to induce the formation of a laser-induced periodic surface structure (LIPSS) on the surface of PET film, and prepared nano grating templates with a period of about 450 nm and a depth of about 108 nm. The LIPSS-PET template is hydrophilic (contact angle is about 79°) due to the photooxidation effect (formation of carboxyl groups) produced during laser processing. The researchers further used their original 3D-printing-assisted nanoimprint (3DPrANIL) technology to accurately copy the morphology of the template onto the polycaprolactone (PCL) film, as shown in Figure 5 [182]. It is noteworthy that the PCL replica exhibits enhanced hydrophobicity due to its inherited nanostructured morphology, and its water contact angle increases from 80° of unstructured PCL to 104°, successfully realizing the functional transfer from hydrophilic template to hydrophobic replica [182]. This study not only expands the application boundary of PET in the preparation of functional templates, but also provides a new idea for the preparation of nanostructured surfaces in a low-cost, non-clean room environment [182].

Based on the above research, the core advantages of PET-based nanoimprinted film can be summarized as follows: (1) excellent optical transmission and mechanical flexibility make it an ideal substrate for flexible optoelectronic devices; (2) good compatibility with hot embossing and UV embossing processes, and relatively wide process window in T-NIL; (3) the surface energy can be effectively regulated by surface treatment (corona, plasma) to improve the wettability with UV resin. However, its limitations also deserve attention: (1) the sensitivity of viscoelasticity to temperature leads to a wide process window but needs to be accurately controlled; (2) there are still challenges in the filling of microstructure with aspect ratio (>0.625), which need to be improved through the collaborative optimization of temperature and packing time; (3) in RTR-UV-NIL, the interface interaction between RTR-UV-NIL and UV resin needs to be accurately matched to prevent bubble entrapment defects. At present, PET nanoimprint film has been widely used in the fields of flexible optoelectronic devices [183] (organic photovoltaic, OLED), microfluidic chips [184], micro optical elements [185] (microlens array, diffraction grating), super hydrophobic/antifouling surfaces [186] and high-precision anti-counterfeiting identification [187], and is developing in the direction of higher efficiency, lower cost, all green solvent treatment and multi-functional integration. In the future, the combination of in situ monitoring technology and machine learning-aided process optimization is expected to further break the performance limit of PET in the replication of high-precision, high-aspect-ratio micro nano structures, and expand its application in emerging fields such as wearable electronics, biosensors and intelligent packaging.

### 2.3. Research Progress on PVA-Based Nanoimprint Film

**Figure 6:**
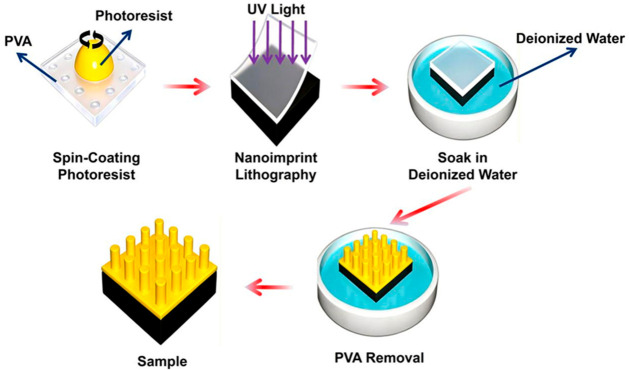
Schematic diagram of nanoimprint lithography process using PVA as sacrificial mold [61].

In nanoimprint lithography technology, polyvinyl alcohol (PVA) has unique water solubility, good film-forming property and adjustable physical and chemical properties. At present, two main research and application directions have been developed: one is as a water-soluble sacrificial die material, and the other is as an imprint structural material with an environmental response function. As a sacrificial mold, the core advantage of PVA is that its water solubility can completely eliminate the shear stress caused by mechanical demolding in the traditional nanoimprint process, so as to avoid collapse or fracture of high-aspect-ratio nanostructures in the separation process, as shown in Figure 6 [61,188]. For example, researchers successfully prepared a defect-free dielectric super lens with an aspect ratio of up to 5.8 using a PVA replica mold combined with a wet etching process, which achieved centimeter-level high-fidelity pattern transfer [61]. At the same time, in the early stage, the stripping process based on PVA monolayer and hydrosilsesquioxane/PVA bilayer was developed. Using water as the developing and stripping solvent, an environmentally friendly metal patterning scheme was constructed, and the gold nanostructure with a linewidth of 100 nm was successfully obtained [61]. However, when PVA is used as a sacrificial layer, its low etching resistance and difficulty in forming an ideal suspended structure in the single-layer process limit its application accuracy in metal stripping with small linewidth (<500 nm). In order to make up for this deficiency, the researchers developed a double-layer structure composed of high-anti-etching materials such as HSQ. Using HSQ as the upper hard mask for pattern transfer, the bottom layer of PVA was isotropically over-etched by O2 plasma to form an inverted conical profile conducive to stripping, so as to promote the linewidth of the gold structure to 100 nm [61].

On the other hand, as a humidity responsive hydrogel, PVA has been widely used in the construction of tunable optical devices in recent years. The physical mechanism is that PVA film can absorb or release water molecules according to the change in environmental relative humidity, resulting in significant volume swelling (the volume change rate can reach 62.5%), so as to dynamically adjust the physical size of the optical resonator [189]. Based on this principle, researchers constructed a Fabry–Perot etalon structure composed of disordered silver nanoparticles/PVA/aluminum mirror. In this study, the porous characteristics of the disordered silver nanoparticle layer were used to accelerate the penetration of water molecules, achieving rapid response at the millisecond level, and RGB full-color pixels with a resolution of up to 700 nm were prepared by 3D nanoimprint technology, showing the application potential in the field of dynamic display and humidity sensing [189]. It is worth noting that although the swelling characteristics of PVA endow its active tuning function, its hygroscopicity may cause distortion in precision-patterned applications requiring high dimensional stability (such as the metal stripping process), so it is often necessary to introduce hydrophobic treatment or a compound with other materials for regulation [190].

To sum up, the research of PVA-based imprint film is evolving from a single water-soluble sacrificial layer to a structural material with an integrated environmental response function. As a sacrificial die, it has significant advantages in realizing stress-free demolding and high fidelity replication of a high-aspect-ratio structure. It is one of the key materials to promote the development of nanoimprint lithography to green and high-precision manufacturing. As a functional material, its exploration in humidity sensing, dynamic display and other fields further expands the application boundary of nanoimprint technology in intelligent photonic devices. How to balance the processability and inherent hygroscopicity of PVA, and further improve its structural stability in the transfer of high-precision graphics, is still the focus of current research and the direction of future development.

### 2.4. Comparative Overview of Single-Polymer Imprint Films

Table 3 summarizes the key properties of PDMS, PET, and PVA as single-component imprint materials, revealing distinct trade-offs. PDMS exhibits a low Young’s modulus, enabling conformal contact and easy demolding, but its softness leads to deformation under external pressure, limiting ultimate resolution. Its high visible transmittance (>98%) and room temperature curing offer excellent compatibility with UV-NIL, while durability exceeding 100 imprints with a proper resist selection is achievable. PET, by contrast, is mechanically rigid and dimensionally stable, supporting continuous roll-to-roll processing with stable performance over 50 rolls. However, its higher curing temperature (~80 °C) and hard surface can cause substrate damage during demolding. PVA combines high stiffness with room temperature processing and top visible transparency (>98%), and uniquely provides water-sacrificial capability for stress-free release, enabling aspect ratios of up to 5.8. Yet it cannot be reused as a sacrificial mold and is susceptible to humidity-induced swelling (≈62.5% volume change). In essence, PDMS favors flexibility and reusability but compromises dimensional accuracy; PET balances production scalability with surface hardness; PVA offers the highest-precision release but lacks durability.

## 3. Polymer Composite Nanoimprint Film

The core of nanoimprint lithography technology is to realize the transfer of graphics from template to substrate through physical contact. This process puts forward complex and mutually restricted requirements for the mechanical properties of imprint film materials. The ideal imprint film needs to have enough flexibility in the imprint stage to achieve conformal contact with the substrate [191]. It can realize non-destructive peeling after the pattern curing, and maintain high dimensional stability during the pattern-transfer process [192]. However, it is often difficult for traditional single-polymer materials to meet these requirements at the same time, which prompted researchers to explore the construction of a multifunctional integrated composite imprint film through the composite and lamination of different polymers.

### 3.1. Limitations of Single-Polymer Materials

At present, the mainstream imprint film materials are mainly divided into two categories: elastic materials and flexible materials, each with show unique advantages, but which also have inherent defects that are difficult to overcome [193,194]. This distinction is rooted in their intrinsically different mechanical behavior, rather than in their macroscopic deformability alone. Elastic materials, typified by PDMS, behave in a manner analogous to human skin—they can be stretched reversibly under external load, and their microscopic surface exhibits a very low mechanical modulus, meaning that the local stiffness is small [195]. In contrast, flexible materials such as PET and PVA are more akin to paper—they can be readily bent or folded yet cannot undergo substantial tensile elongation. While such materials display macroscopic flexibility, their surface hardness is relatively high, which tends to generate considerable localized stress upon contact with a counterpart [196]. In order to reflect this intrinsic difference in mechanical response, PDMS is categorized as an elastic material, whereas PVA and PET are designated as flexible materials throughout this article.

**Figure 7:**
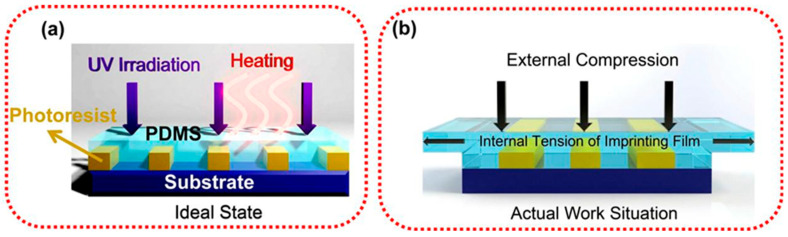
(**a**) Nanoimprint lithography process in an ideal state; (**b**) Nanoimprint lithography process in the actual situation [34].

Elastic materials such as polydimethylsiloxane (PDMS) have been widely used in the field of nanoimprinting due to their low Young’s modulus, excellent flexibility, low surface energy and good chemical stability. A PDMS material can achieve close conformal contact with the substrate without external pressure or low pressure, and the stripping process is relatively easy, with less damage to the template and substrate, making it an ideal choice for the preparation of high-precision nanostructures such as the patterned sapphire substrate. However, the elastic properties of PDMS bring significant problems in practical applications. As shown in Figure 7a, nanoimprint lithography can complete 1:1 perfect replication in an ideal state, but the actual situation of PDMS as a nanoimprint film is shown in Figure 7b. In the imprint process, the external extrusion force will generate internal tension of the PDMS film, resulting in its longitudinal thinning and transverse expansion. This kind of stress deformation will directly lead to the change in the size of the transferred pattern, causing linewidth fluctuation and pattern distortion, which limits its application in sub-100 nanometer-scale high-precision machining [34].

**Figure 8:**
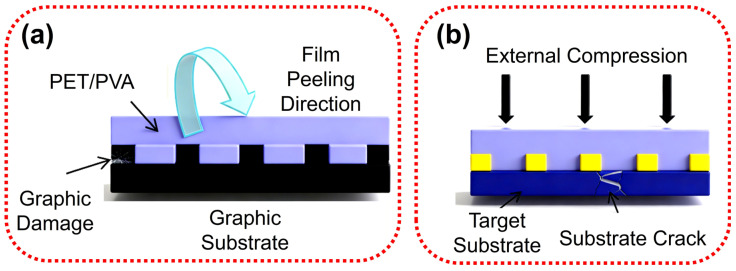
Schematic diagrams of damage caused by flexible nanoimprint films: (**a**) damage to the graphics substrate during peeling due to strong adhesion after curing; (**b**) damage to the target substrate during imprinting due to direct pressure transmission without buffering capacity [34].

In contrast, the flexible materials represented by polyethylene terephthalate (PET) and polyvinyl alcohol (PVA) can effectively overcome the deformation problem of elastic materials due to their high hardness and excellent dimensional stability. These hard materials can accurately transfer the graphics to the target substrate in an ideal ratio of 1:1 during the imprinting process, which significantly improves the accuracy of graphics transmission. However, flexible materials also face severe challenges. First of all, as shown in Figure 8a, high hardness materials such as PET/PVA have strong adhesion with the graphics substrate after curing, which makes it difficult to peel off and may even damage the graphics substrate. Secondly, as shown in Figure 8b, hard materials lack buffering capacity during the imprinting process, which will transmit all the external pressure to the target substrate, and it is very easy to damage the brittle or precision substrate [34].

To sum up, a single polymer material has formed an irreconcilable contradiction between “elasticity and deformation” and “hardness and peeling”, which provides a clear demand orientation for the research of composite imprint film.

### 3.2. Elastomer–Rigid Bilayer Composite Template

**Figure 9:**
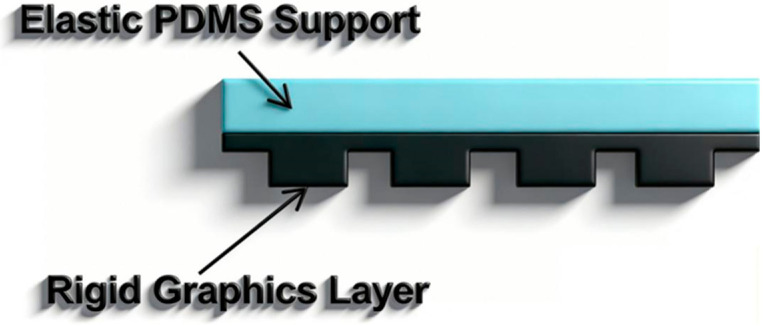
Schematic diagram of nano imprint composite film structure [83].

In order to integrate the high resolution of nano imprinting and the flexibility of soft imprinting, researchers developed a double-layer composite template composed of an elastic support layer and a rigid structure layer. The core idea of this design is to separate the graphical function from the mechanical adaptation function. The composite template proposed by the research team of Nanjing University is a representative work in this direction. The template is composed of a PDMS elastic support layer at the lower layer and a rigid structural layer of ultra-thin and highly crosslinked UV-curable material at the upper layer, as shown in Figure 9 [83]. The elastic support layer gives the template the ability to closely adhere to the planar and curved substrates without external pressure, while the rigid structure layer provides sufficient mechanical strength to ensure the accurate imprinting of sub-15 nanometer high-resolution graphics.

The interface between the two layers is the key technical difficulty of the structure. In order to solve the problem that PDMS and rigid materials are prone to—delamination due to large modulus differences—the team developed the polymer interpenetrating network structure bonding technology. In the preparation process, the PDMS elastic layer pre-absorbs the UV-curable resin monomers. When curing, these monomers participate in the crosslinking reaction of the rigid layer at the same time, and penetrate each other with the PDMS network to form a gradient interface, so as to realize the solid combination of the two layers of materials. In addition, the rigid film was prepared on the elastic polymer substrate by the controllable folding method, and the ordered nano/micron grating was formed by stress induction, which provided a new way for the preparation of a low-cost and large-area composite template.

This elastic rigid double-layer composite structure successfully combines the advantages of the two materials, which not only realizes high-resolution graphics transfer, but also has excellent conformal contact ability, and can be extended to curved substrates. However, its rigid structural layer still has the risk of cracking when it is highly bent, and its tolerance to small particles in the imprint environment is limited.

### 3.3. Sandwich Structure Composite Imprint Film

**Figure 10:**
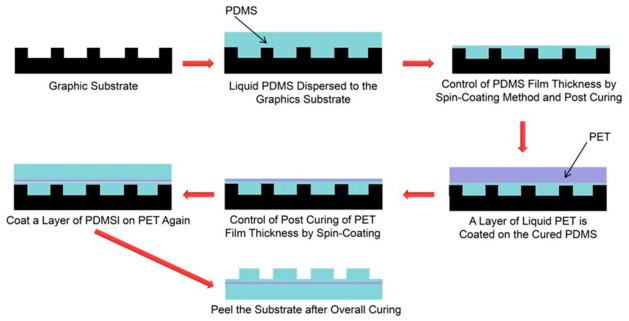
Schematic diagram of preparation process of composite nanoimprint film with “sandwich” structure [34].

In view of the contradiction between the mechanical response of a single material, the researchers further proposed a “sandwich” structure composite imprinted film, which decouples the lateral expansion of the elastic layer and the deformation of the imprinted layer by introducing an intermediate layer. The structure is composed of an upper silica gel protection area, a middle flexible PET substrate and a lower silica gel imprint area. To prepare this composite film, a lower silica gel layer (imprint area), a PET film, and an upper silica gel layer (protection area) are sequentially applied onto the patterned substrate, as shown in Figure 10 [34].

**Figure 11:**
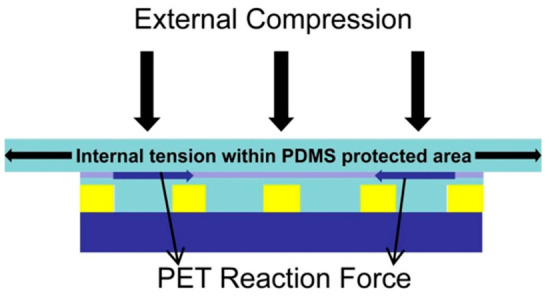
Schematic diagram of the principle of composite nanoimprint film with “sandwich” structure [34].

The core mechanical mechanism of the structure is shown in Figure 11 [34]. When the external extrusion force acts on the silica gel protection zone in the upper layer, the zone generates internal tension and lateral expansion. However, due to the strong contact between the silicone-protected area and the middle-layer PET substrate, the PET substrate has a corresponding reaction force due to its high hardness and dimensional stability, which effectively inhibits the transmission of lateral expansion to the lower-layer silicone imprint area. Therefore, the lower silicone imprint area can maintain its original shape and size, ensuring that the micro nano structure on the graphics substrate can be transferred to the photoresist column of the target substrate with 1:1 high precision. At the same time, the silica gel material in the lower layer still retains its advantages of easy peeling and less damage to the substrate.

The sandwich structure design realizes the coordination and division of mechanical functions. The upper layer of silica gel provides protection and bears external forces. The middle layer of PET resists transverse tension and maintains structural stability. The lower layer of silica gel is responsible for high fidelity graphics transfer and easy peeling. The structure effectively solves the deformation problem of a single elastic material, the peeling difficulty of a single flexible material and the substrate damage problem, and provides a new solution for high-precision and high-reliability nano imprinting.

### 3.4. Theoretical Insights into Interface Reliability of Composite Imprint Films

The structural integrity of both bilayer and sandwich composite films relies heavily on the reliability of their internal interfaces. In the elastic–rigid bilayer architecture, a sharp modulus mismatch between the compliant PDMS support and the ultra-thin, highly cross-linked rigid layer would otherwise concentrate interfacial stress, causing progressive delamination under cyclic loading. The interpenetrating polymer network (IPN) bonding strategy mitigates this problem by establishing a gradient–modulus transition zone rather than an abrupt material boundary. As the UV-curable resin monomers swell into the PDMS surface and subsequently cross-link, a molecularly entangled region is formed in which the two networks become mechanically interlocked. This entanglement provides both physical anchoring and a distributed stress transfer path; external loads are spread across a diffuse interfacial volume instead of being concentrated along a discrete two-dimensional plane. Consequently, the peak shear stress is substantially reduced, and the energy required for interfacial crack propagation is increased, which explains the enhanced delamination resistance observed experimentally.

In the sandwich structure, the middle PET layer functions as a mechanical constraint that suppresses the lateral expansion of the adjacent elastic PDMS layers through a Poisson coupling mechanism. When a normal compressive load is applied, the near-incompressibility of PDMS (Poisson’s ratio ≈ 0.5) generates a tendency for transverse expansion. The high-modulus PET core, tightly bonded to the PDMS layers, produces an opposing elastic restoring force that resists this expansion. This constraint effectively decouples the macroscopic deformation of the stamp from the microscopic pattern fidelity in the lower imprint region, thereby preserving the dimensions of the transferred nanostructures. In addition, the symmetric sandwich layout balances deformation fields across the thickness, minimizing the bending and warpage that would arise from an asymmetric stress distribution. The adhesion at the PDMS/PET interface is essential to this function; any interfacial slip would undermine the constraint and reintroduce pattern-shift errors.

### 3.5. Application Fields of Composite Imprint Film

Polymer composite imprinted films have shown potential applications in many micro nano manufacturing fields [197,198,199]. In the fabrication of graphical sapphire substrates, the composite template realizes the low-cost and large-area preparation of a high-uniformity micro nano structure, which significantly improves the light extraction efficiency of the LED chip. In the field of curved surface devices, the flexibility of the composite template enables it to achieve high-resolution pattern transfer on non-splanar substrates such as fiber cylindrical surfaces and artificial compound eyes. In the manufacture of optoelectronic devices, metal gratings, photonic crystal structures and other devices are fabricated through the combination of composite templates, decompressive printing adhesives, double-curing transfer layers and other material systems.

### 3.6. Chapter Summary

The research of polymer nanocomposite nanoimprint films has experienced structural evolution from single-material to multi-functional synergy. The elastic rigid double-layer composite template realizes the preliminary integration of a high resolution and shape-preserving by separating the graphical function and mechanical adaptation function; the sandwich structure composite imprinted film can further solve the contradiction between elastic deformation and peeling performance by introducing an intermediate layer to decouple the mechanical response. Current research has confirmed that the advantages of each component material can be brought into full play and the limitations of a single material can be effectively avoided through the stacking and compounding of different polymers. However, the composite structure still faces challenges in interface reliability, multi-layer process compatibility and large-scale manufacturing stability. Future research needs to further explore new polymer combinations, optimize interface bonding technology, and develop matching process systems to promote the application of composite imprint films in higher-precision, more complex structures, and more large-scale production.

## 4. Polymer/Transparent Electrode Composite System

Nanoimprint lithography (NIL) technology puts forward increasingly stringent requirements for imprint film materials [200,201,202]. Although traditional single-polymer films (such as PDMS, PVA, PET, etc.) have their own advantages in flexibility, transparency or demolding, they are often limited by their inherent functional simplicity in practical applications, such as bottlenecks in thermal management, electrostatic dissipation, etc. [101,111,203,204]. In order to break through these limitations, the functional transparent electrode materials (such as silver nanowires) are compounded with a polymer matrix to construct a multilayer or embedded structure, which has become the frontier direction of the current research on NIL imprint film materials. This composite structure aims to realize the integration and optimization of optical, electrical, thermal, mechanical and other properties through the synergistic effect of each component, and provides an innovative path to solve the limitations of traditional imprinted films. The core advantage of polymer/transparent electrode composite films is that by introducing high-conductive networks such as AgNWs, it gives the originally insulated polymer matrix a new electric heating or conductive function, so as to accurately meet the specific challenges in the NIL process.

### 4.1. Thermal Management

**Figure 12:**
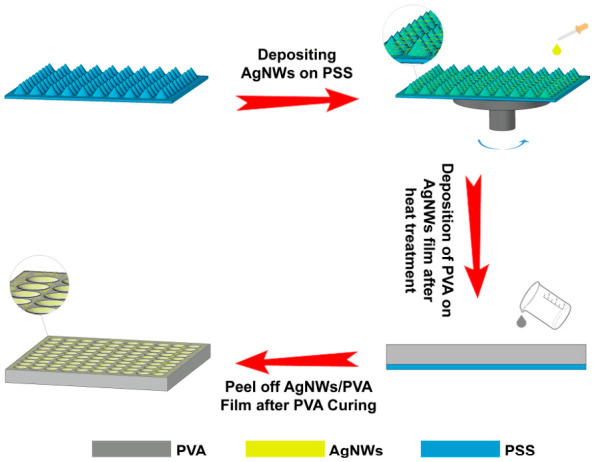
Preparation process of AgNWs/PVA composite nanoimprint film [98,101].

**Figure 13:**
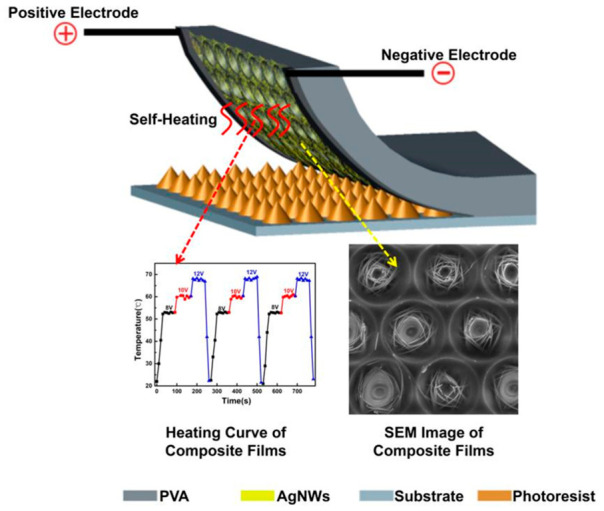
Heating principle of self-heating composite nanoimprint film [101].

In terms of thermal management, researchers have developed a self-heating film (AgNWs/PVA) based on the composite of AgNWs and polyvinyl alcohol (PVA). The specific preparation process is shown in Figure 12 [98,101]. In this study, the Joule heating effect of the AgNWs network was used to realize the rapid and uniform heating of the imprinted film itself. The innovation is that the heat source is directly integrated into the imprinted film, abandoning the traditional external hot plate or infrared heating method, as shown in Figure 13 [101]. It is worth noting that the areal density of AgNWs, governed by the number of spin-coating layers, exerts a coupled influence on the electrical and optical performance of the composite film. As the number of AgNWs layers increases, the sheet resistance progressively decreases owing to the formation of a denser conductive network. However, this simultaneously leads to a gradual decline in UV transmittance, as a higher density of nanowires introduces greater light scattering and absorption. To satisfy the dual requirements of sufficient Joule heating and adequate UV exposure during NIL, a balance must be struck; the sheet resistance is controlled at approximately 10 Ω/sq while maintaining a UV transmittance of 84.82% at 248 nm, which corresponds to the optimized deposition condition. The experimental results show that the composite film can be heated to 60 °C in 30 s when 10 V of voltage is applied, and cooled to room temperature in 20 s after power failure. Its heating rate (1.27 °C/s) and cooling rate (1.90 °C/s) are much higher than those of the traditional hot plate. This ultra-fast temperature rise and fall ability not only significantly improves the efficiency of the NIL process, but also realizes the accurate control of heating temperature by adjusting the voltage, which helps to improve the accuracy of pattern transfer.

### 4.2. Electrostatic Control

**Figure 14:**
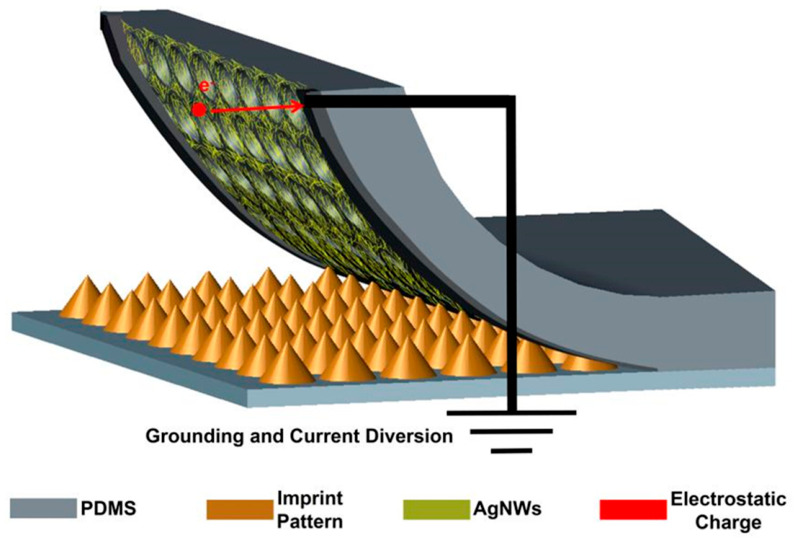
Antistatic principle of composite nanoimprint film [111].

In terms of electrostatic control, the research focuses on solving the problem of electrostatic accumulation caused by friction electrification in the NIL process [205,206,207,208]. A three-dimensional transparent conductive film composed of AgNWs and polydimethylsiloxane (PDMS) was proposed. PDMS is a common elastic embossing template material in NIL, but its high insulation makes static electricity unable to be released, which is easy to cause pattern distortion or equipment damage. In this study, AgNWs and PDMS were spin-coated on the patterned substrate in turn, and then peeled after curing. The AgNWs conductive network was successfully embedded into the PDMS surface. In the AgNWs/PDMS system, the individual silver nanowires possess a diameter of approximately 30 nm and a length of about 25 μm, yielding a high aspect ratio that facilitates the formation of a percolated conductive network at relatively low areal density. The transmittance of the composite film is up to 90% under 325 nm UV light, and the sheet resistance is about 20 Ω/sq. The moderate conductivity provides a channel for rapid dissipation of surface electrostatic charge. From the perspective of mechanism, the introduction of the AgNWs network has increased the effective conductivity of the composite by several orders of magnitude, reducing the charge relaxation time from the range of minutes to hours of pure PDMS to the millisecond level, thus realizing instant electrostatic neutralization during the imprinting process, significantly reducing the surface charge density and avoiding electrostatic interference (as shown in Figure 14 [111]). This study not only shows the advantages of the AgNWs/PDMS composite film in eliminating static electricity, but also its self-supporting three-dimensional microstructure surface proves the ability of the composite system to accurately copy high-precision patterns.

### 4.3. Synergistic Advantages of Composite Structures

The above research clearly shows the synergistic effect of the polymer/transparent electrode composite system. On the one hand, the AgNWs network, as a functional phase, endows the polymer matrix with key properties such as conductivity and self-heating. On the other hand, the polymer matrix (such as PVA, PDMS) is used as a protective layer and structural layer, which improves the overall stability and processability of the composite film. For example, the close wrapping of PVA effectively isolated the contact between AgNWs and air, so that it could still maintain resistance stability in the exposure test for up to one year, and significantly enhanced the antioxidant capacity of AgNWs. Similarly, the elastic matrix of PDMS not only ensures that the AgNWs/PDMS composite film can maintain good conductivity after repeated stretching (more than 100 times of 40% stretching) (the sheet resistance only increases by about 12%), but also lays a foundation for its application in the field of flexible electronics.

However, the design of composite systems also faces performance tradeoffs. In the AgNWs/PVA system, increasing the number of layers of AgNWs in order to pursue lower sheet resistance (i.e., stronger heating performance) will lead to a significant decrease in UV transmittance. Therefore, researchers must find the optimal balance between heating efficiency (conductivity) and light transmittance. For example, by optimizing the number of spin-coating layers, the sheet resistance is controlled at about 10 Ω/sq on the premise of ensuring that the 248 nm light transmittance is more than 80%, so as to meet the comprehensive requirements of the NIL process. In the AgNWs/PDMS system, tensile deformation will lead to the thinning of the geometric structure of the AgNWs network, and even local fracture, resulting in the increase in resistance. This indicates that while pursuing high tensile properties, it is necessary to optimize the initial network density of AgNWs and the interface adhesion with PDMS to maintain stable electrical properties in a larger strain range.

### 4.4. Application Fields of Composite Film

At present, the research on polymer/transparent electrode composite imprinted film has shown a clear practical potential. Based on its unique performance combination, these materials are mainly used in the following fields: (1) high-precision and high-efficiency NIL process, such as AgNWs/PVA self-heating, where the film can be directly used as a heating imprinting template, simplifying the equipment structure and improving the temperature rise and fall cycle efficiency, which is suitable for UV nano imprinting or the thermal imprinting process requiring rapid thermal cycles. (2) NIL manufacturing sensitive to static electricity, such as the AgNWs/PDMS conductive film, can be used as an anti-static imprinting template or intermediate layer, which can effectively suppress static defects and improve yield in the manufacturing process of optoelectronic devices, high-density storage media and other strict requirements on static electricity. (3) Flexible optoelectronic devices; two kinds of composite films show good flexibility and mechanical stability, which can be further extended to the roll nanoimprinting process in the fields of flexible displays, wearable sensors and so on.

Despite significant progress, several challenges remain in this area. How to further improve conductivity, heating efficiency or anti-static ability without sacrificing transparency and flexibility requires more detailed material design and structural engineering. Although the short-term and medium-cycle stability tests performed well, life under complex working conditions (such as high humidity, high temperature, and long-term continuous operation) still needed to be systematically evaluated. Finally, the exploration of a low-cost and large-scale preparation process. At present, most of the reported laboratory processes such as spin-coating and peeling are used. How to transform them into an industrial production process compatible with roll-to-roll production and controllable uniformity is the key to promote the practical application of this technology.

In summary, the polymer/transparent electrode composite system successfully introduced self-heating, antistatic and other key properties for nanoimprint films by combining functional materials such as AgNWs with polymer matrices such as PVA and PDMS, effectively overcoming the limitations of traditional single-polymer materials. This design concept of functional integration and performance synergy has opened up a new path for the development of the next generation of high-performance, multi-functional nanoimprint film materials, and is expected to promote the application of NIL technology in a wider range of advanced manufacturing fields.

## 5. Conclusions

To sum up, the research of imprint film materials used in nanoimprint lithography has experienced a profound evolution from single-component polymer to composite structure, from passive adaptation to active functional integration. PDMS, PET, PVA and other single polymers have their own unique advantages. PDMS provides low surface energy and conformal contact, PET provides high transparency and low-cost roll-to-roll compatibility, and PVA realizes stress-free demolding through water solubility. However, their inherent limitations, including elastic lateral expansion (PDMS), difficult release and substrate damage (PET), and moisture-induced expansion (PVA), have promoted the development of composite structures. The elastomer–rigid bilayer template successfully separates the patterning and mechanical compliance functions, while the sandwich structure membrane decouples the mechanical response by introducing an intermediate layer, which further solves the trade-off between elastic deformation and peeling performance. Recently, polymer/transparent electrode hybrid systems, such as the AgNWs/PVA self-heating film and AgNWs/PDMS antistatic film, have shown significant functional integration, which can achieve rapid Joule heating and millisecond charge dissipation. These advances have effectively solved the key challenges in thermal management and electrostatic control that cannot be achieved by traditional materials. Despite these achievements, there are still challenges in improving the interface reliability of multilayer structures, balancing the electrical/optical properties and expanding the manufacturing scale. Moreover, it is worth noting that, with the exception of single-polymer imprint films, most composite systems, including multilayer polymer films and polymer/transparent electrode hybrid films—remain largely at the laboratory stage, while systematic engineering practice and commercial-scale production validation are still lacking. Critical issues such as large-area uniformity control, long-term operational stability under realistic processing conditions, and cost-effective compatibility with roll-to-roll manufacturing have yet to be adequately addressed. Future efforts should focus on new polymer combinations, robust bonding technologies and industry-compatible processes. This review emphasizes the importance of collaborative material design, and provides a roadmap for the development of next-generation imprint films that meet the requirements of high-precision, high-throughput and multifunctional nanoimprint lithography.

## Figures and Tables

**Table 1:** Changes in film properties after more than 100 nanoimprints [161].

Number of Imprints	SU-8 2002 Relative Hardness (Average, Range)	Reference Relative Hardness (Average, Range)
10–20	(1.1, 1.0–1.2)	(0.95, 0.8–1.1)
20–40	(0.9, 0.8–1.0)	(1.0, 0.9–1.1)
40–60	(1.4, 1.3–1.5)	(1.0, 0.8–1.2)
60–80	(0.95, 0.8–1.1)	(0.9, 0.5–1.6)
80–100	(1.1, 0.9–1.2)	(1.2, 1.0–1.3)
100–120	(1.1, 1.0–1.2)	(1.2, 1.1–1.3)

**Table 2:** Comparison of aspect ratio between simulation and experimental results [171].

Designed Line Width (μm)	Mold Aspect Ratio	Simulation of PET Aspect Ratio	Experiment of PET Aspect Ratio
0	2.30	0.35	0.65
80	1.30	0.35	0.60
120	0.85	0.35	0.58
160	0.75	0.38	0.68
200	0.55	0.38	0.52
240	0.48	0.35	0.42
280	0.40	0.32	0.38
320	0.35	0.28	0.33

**Table 3:** Comparison of key properties of PDMS, PET, and PVA single-material imprint films.

Material	Young’s Modulus	Transmittance	Curing Temperature	Max. Reported Resolution	Durability/Reusability	Other Features
PDMS	Low	>98% in visible range	room temperature	~100 nm (direct etch with PS mask)	>100 imprints	Compliant conformal contact; Easy demolding; Deformation under external pressure
PET	High	>87% in visible range	~80 °C	~300 nm (RTR-UV-NIL)	Stable over 50 rolls in continuous RTR operation	Bendable but not stretchable; Relatively hard surface
PVA	High	>98% in visible range	room temperature	100 nm (Au pattern, bilayer with HSQ); aspect ratio up to 5.8 (metalens)	Not reusable as a sacrificial mold	Water-soluble for stress-free demolding; Humidity-induced volume swelling ≈ 62.5

## Data Availability

No new data were created or analyzed in this study.

## Abbreviations

The following abbreviations are used in this manuscript:

NIL	Nanoimprint lithography
PDMS	Polydimethylsiloxane
PET	Polyethylene terephthalate
PVA	Polyvinyl alcohol
AgNWs	Silver nanowires
R2R	Roll-to-roll
UV-NIL	Ultraviolet nanoimprint lithography
HSQ	Hydrogen silsesquioxane
RGB	Red, green, blue
SU-8	Epoxy-based negative photoresist
LED	Light-emitting diode
T-NIL	Thermal nanoimprint lithography
RTR-UV-NIL	Roll-to-roll ultraviolet nanoimprint lithography
VOF	Volume of fluid
OC	Open channel
IS	Imprinting speed
OSC	Organic solar cell
R2P	Roll-to-plate
JSC	Short-circuit current density
FF	Fill factor
PCE	Power conversion efficiency
ITO	Indium tin oxide
PBDB-T	Poly[4,8-bis(5-(2-ethylhexyl)thiophen-2-yl)benzo[1,2-b:4,5-b′]dithiophene-2,6-diyl]-alt-(4-(2-ethylhexyl)-3-fluorothieno[3,4-b]thiophene-)-2-carboxylate-2-6-diyl]
ITIC	3,9-bis(2-methylene-(3-(1,1-dicyanomethylene)-indanone))-5,5,11,11-tetrakis(4-hexylphenyl)-dithieno[2,3-d:2′,3′-d′]-s-indaceno[1,2-b:5,6-b′]dithiophene
LIPSS	Laser-induced periodic surface structure
PCL	Polycaprolactone
3DPrANIL	3D printing-assisted nanoimprint lithography
